# Contribution of birth weight and adult waist circumference to cardiovascular disease risk in a longitudinal study

**DOI:** 10.1038/s41598-017-10176-6

**Published:** 2017-08-29

**Authors:** Jingyan Tian, Miaoyan Qiu, Yanyun Li, Xuan’e Zhang, Haiyan Wang, Siming Sun, Nora Sebeca Sharp, Wenxin Tong, Hailuan Zeng, Sheng Zheng, Xiaomin Song, Weiqing Wang, Guang Ning

**Affiliations:** 1 National Clinical Research Center for Endocrine and Metabolic Diseases, Shanghai Institute of Endocrine and Metabolic Diseases, Department of Endocrinology and Metabolism, Ruijin Hospital, Shanghai Jiao Tong University School of Medicine, Shanghai, China; 2 Department of Diabetes Complications and Metabolism, Beckman Research Institute of City of Hope, Duarte, CA USA; 3 Division of Non-communicable Diseases and Injuries, Shanghai Municipal Center for Disease Control and Prevention, Shanghai, China; 4 Department of Endocrinology and Metabolism, Yangpu Hospital, Tongji University School of Medicine, Shanghai, China; 5 Ping Liang Community Health Service Center, Yang Pu district, Shanghai, China

## Abstract

To determine the association of birth weight (BW) and waist circumference (WC) on cardiovascular disease (CVD). The longitudinal cohort study consisted of 745 participants who were able to provide their birth weight information and were followed from 2002 to 2014. During the follow-up, 83 events of CVD were confirmed. After adjusting for confounding factors, subjects with birth weight <2500 g were at a significantly increased CVD risk when compared to subjects with birth weight between 2500–3999 g (OR 2·47, 95%CI, 1·07–5·71). When high waist circumference (HWC), a measurement of adult obesity, was incorporated into stratifying factors according to presence or absence of low birth weight (LBW, birth weight <2500 g), adjusted CVD risk was significantly elevated in -LBW/+ HWC group (OR 1·94, 95%CI, 1·10–3·43) and marginally significantly increased in +LBW/-HWC group (OR 2·94, 95%CI, 1·00–8·64). CVD risk was highest in subjects with LBW and HWC (+LBW/+HWC), OR 4·74 (95%CI, 1·48–15·21). Higher waist circumference in adulthood is an especially strong risk factor for cardiovascular disease among those small at birth. In this cohort, birth size and adiposity in adulthood interact to predict events of cardiovascular disease.

## Introduction

Accumulating evidence indicates that early life determinants may be important in the pathogenesis of adult disease^1–5^. In 1989, birth weight (BW) was reported to be inversely associated with the risk of dying from ischemic heart disease during adulthood^6^. Since then low birth weight (LBW) has been consistently confirmed as a risk factor of cardiovascular disease (CVD) by several independent studies^3, 4^. In the meantime, others carried on to investigate anthropometric measurements in adulthood to find better predictors^7, 8^. Some researchers believe that these two factors should not be treated independently, and that BW and adult body size are likely to interact with each other to increase the risk of coronary heart disease^4^. It was reported that waist circumference (WC) was the best predictor for CVD among the anthropometric measurements in population^9^. However, no thorough investigation has been done regarding the comparison and combination of WC with other early life risk factors. The present study investigated the effects of BW and WC on CVD risk by analyzing a cohort in a Chinese community with 10 years follow-up.

## Results

At baseline, a total of 1010 individuals provided their BW information from birth certification and hospital case records. Among them, 745 participants (283 males and 462 females) were followed and were enrolled in this study. The mean follow-up time was 10·9 years (10·5y–12·0y). From 2002 to 2014, we confirmed 83 cardiovascular events.

The mean values of anthropometric data and metabolic parameters in 2002 were presented in Table 1 according to BW subgroups. Within this cohort, significant discrepancies were demonstrated among groups concerning gender distribution, WC, incidence of diabetes, baseline glycemic level, cigarettes use, while incidence of hypertension, baseline blood pressure, lipid level, alcohol use and economic status were similar between groups. Furthermore, because about 1/4 individuals were lost to follow up, we compared the participants who attended follow-up with those who were lost and found no significant differences in BW, glucose levels, blood lipid profile, blood pressure and any other factors between these two groups (data not shown).

**Table 1:** Characteristics of the study participants, according to birth weight subgroups at baseline.

Variable	Birth weight category			*P value*
	<2500 g (*N* = *45*)	2500–3999 g (*N* = *659*)	≥400 0 g (*N* = *41*)	
Age (yr)	45.5 ± 8.2	45.8 ± 11.0	43.0 ± 15.2	—
Males (%)	31.1	36.9	63.4	**
Mean BW (g)	2140.0 ± 329.9	3042.3 ± 303.9	4203.7 ± 474.1	**
Waist circumference (cm)	81.2 ± 11.5	78.4 ± 9.7	83.1 ± 9.8	**
Diabetes (%)	28.9	8.8	4.9	**
Fasting glucose (mmol/l)	6.5 ± 2.5	5.7 ± 1.6	5.3 ± 0.7	**
2-h glucose (mmol/l)	6.6 ± 4.2	5.4 ± 2.3	5.7 ± 1.6	**
Hypertension (%)	53.3	40.4	39.0	—
SBP (mmHg)	125.1 ± 16.1	124.3 ± 17.6	125.1 ± 16.0	—
DBP (mmHg)	82.2 ± 11.1	81.3 ± 10.8	81.8 ± 11.0	—
HDL-c (mmol/l)	1.5 ± 0.5	1.4 ± 0.4	1.3 ± 0.4	—
LDL-c (mmol/l)	2.8 ± 0.8	2.7 ± 0.8	2.6 ± 0.9	—
Total cholesterol (mmol/l)	5.0 ± 1.1	4.7 ± 0.9	4.6 ± 1.0	—
Triglycerides (mmol/l)^#^	1.2(0.8–1.6)	1.1(0.7–1.7)	1.2(0.8–1.8)	—
Current smoking (%)	13.3	21.3	41.5	*
Current drinking (%)	13.3	13.4	17.1	—
Active physical activity (%)	17.8	25.5	27.5	—
Economic status (%)				—
High	17.3	6.7	7.3	
Medium	62.1	62.2	73.2	
Low	20.6	31.1	19.5	

Results are given mean ± SD or n (%).

P for difference: −: Non-significant; *p < 0.05; **p < 0.01.

^#^Value of triglycerides did not follow Gaussian distribution, thus medium and interquartile ranges were used instead of mean and standard deviation to describe central and discrete tendency.

During the follow up, 83 (11·1%) events of CVD were confirmed through the health records. The corresponding incidence rates of CVD in BW < 2500 g group, BW among 2500–3999 g group, and BW **≥**400 0 g group were 24·4%, 9·9% and 17·1%, respectively. As shown in Table 2, risk of CVD was lowest in the BW among 2500 g –3999 g group (reference group) and was highest with lower BW (adjusted OR 2·73, 95%CI, 1·20 to 6·25). Individuals with BW equaled to or exceeded 4000 g bore similar risks to those in the reference group (adjusted OR 2·31, 95%CI, 0·84 to 6·30). Findings were confirmed in the additional models with BW dichotomized (BW < 2500 g vs. BW **≥**250 0 g), showing adjusted hazard ratio of 2·87 (95% CI 1·28–6·42) in the group with BW < 2500 g.

**Table 2:** Association of birth weight with adult cardiovascular diseases.

	Crude			Multivariable*		
	OR	95%CI	*P*-value	OR	95%CI	*P*-value
Birth weight (three groups)						
<2500 g	2.96	1.43–6.11	0.007	2.73	1.20–6.25	0.021
2500–3999 g	1	Reference		1	Reference	
≥4000 g	1.88	0.80–4.41		2.31	0.84–6.30	
Birth weight (two groups)						
<2500 g	2.82	1.37–5.81	0.005	2.87	1.28–6.42	0.01
≥2500 g	1	Reference		1	Reference	

*Adjusted for adult age, sex, diabetes status, hypertension status, lipid profile (including HDL-c, LDL-c, Triglycerides, Total cholesterol), waist circumference, drinking and smoking status, physical activity at baseline.

Table 3 shows all the significant contributing factors to increased CVD risk in multivariate analyses after adjusted for age, sex, diabetes status, hypertension status, lipid profile, waist circumference, drinking and smoking status, physical activity at baseline in our study. Female, older age, lower high-density lipoprotein cholesterol (HDL-c) and higher total cholesterol (TC) were all related to higher odds of CVD compared with their counterparts. Most importantly, both BW subgroups and WC were significantly associated with risk of CVD. Every 1-centimeter increase in WC was associated with a 3.2% increase in risk for developing CVD.

**Table 3:** Significant contributing factors in multivariate analyses about the relationship between birth weight and CVD (adjusted for confounders at baseline).

	Chi-square	*P*	B	SE	OR	95%CI
Gender	4.471	0.034				
Male (ref.)					1	1.0–1.0
Female			0.627	0.296	1.871	1.047–3.346
Age (years)	9.706	0.021				
≤40 (ref.)					1	1.0–1.0
41–50	7.615	0.006	2.838	1.028	17.074	2.275–128.122
51–60	9.273	0.002	3.170	1.041	23.808	3.095–183.162
${\boldsymbol{ > }}$60	7.511	0.006	2.994	1.093	19.975	2.347–170.028
HDL-c (mmol/l)	11.163	0.001	−1.522	0.455	0.218	0.089–0.533
TC (mmol/l)	8.277	0.004	0.404	0.140	1.498	1.137–1.972
WC (cm)	4.863	0.027	0.031	0.014	1.032	1.004–1.061
Birth weight (g)	7.870	0.020				
${\boldsymbol{ < }}{\bf{2500}}$	5.675	0.017	1.006	0.422	2.733	1.195–6.252
2500–3999 (ref.)					1	1.0–1.0
${\boldsymbol{\ge }}{\bf{4000}}$	2.653	0.103	0.835	0.513	2.305	0.844–6.295
Constant	19.603	0.000	−7.819	1.766	0.000	

^*^Adjusted for adult age, sex, diabetes status, hypertension status, lipid profile (including HDL-c, LDL-c, Triglycerides, Total cholesterol), drinking and smoking status, physical activity at baseline.

Overall prevalence of high waist circumference (HWC) was 19·7% in the present analyses. In order to examine the influence of BW and adult WC on cardiovascular disease, we identified individuals with BW < 2500 g as LBW (+) group, and the others as LBW (−) group. As is shown in Table 4, compared with subjects without LBW and HWC (-LBW/-HWC, reference group), both subjects with isolated LBW (+LBW/-HWC) and adult obesity (−LBW/+HWC) were significantly related to increased risk of CVD, demonstrating adjusted OR of 2·94 (95%CI 1·00–8·64) and 1·94 (95%CI, 1·10–3·43), respectively. Surprisingly, the risk for developing CVD was highest in subjects with LBW and HWC, up to 4·74 times when compared with that in the reference group, and still much higher than that in any of the isolated condition.

**Table 4:** Adjusted OR for CVD according to birthweight and adult body size at baseline.

Subgroups	Number of CVD (%)	Crude			Multivariable*		
		OR	95%CI	*P*	OR	95%CI	*P*
LBW(−)/HWC(−)	47/568 (8.3)	1	Reference	<0.001	1	Reference	0.005
LBW(−)/HWC(+)	25/132 (18.9)	2.59	1.53–4.39		1.94	1.10–3.43	
LBW(+)/HWC(−)	5/29 (17.2)	2.31	0.84–6.33		2.94	1.00–8.64	
LBW(+)/HWC(+)	6/16 (37.5)	6.65	2.32–19.11		4.74	1.48–15.21	

^*^Adjusted for adult age, sex, diabetes status, hypertension status, lipid profile (including HDL-c, LDL-c, Triglycerides, Total cholesterol), drinking and smoking status, physical activity at baseline.

## Discussion

The present analysis demonstrated that subjects with LBW and HWC in adulthood were associated with the increased risk of CVD after controlling for potential confounders. Subjects with LBW and HWC in adulthood are associated with higher risk of CVD, up to 4·74 times when compared with those without LBW and HWC. These findings suggest that both LBW and adult HWC could be simple and practical predictors of unfavorable CVD outcomes in the general population.

BW is inversely associated with CVD risk factors such as raised blood pressure, dyslipidemia, and glucose intolerance^10–13^. But only a small number of studies have demonstrated an association between BW and CVD outcomes, which is considered of greater importance than showing that size at birth is related to risk factors^3, 4, 14^, and even fewer have looked at this association in population-based Chinese study. Our study is based on general population from a wide range of age distribution, and supports the importance of both BW and adult WC in predicting CVD.

Adjustment for adult WC did not change the association between BW and CVD in the present analysis. Subjects with LBW exhibited higher risk for CVD, while subjects with high birth weight (BW ≥ 4000 g) showed similar risk when compared with those with normal BW (2500–3999 g). Our results support a previous analysis that the risk of coronary heart disease increased linearly for subjects with BW below 3·4 kg and reached 1·28 (95% CI: 1·13 to 1·44) with BW at 2 kg. The association weakened with BW above 3·4 kg, and there was virtually no association when subjects with BW from about 4 kg^15^.

In our study, using logistic regression model showed that age, sex, HDL-c and TC at baseline, BW and WC were factors contributing to the development of CVD consistent with many studies^16–19^. Previous investigators suggested that BW and adult body size could not be investigated independently^4^, while comparison and combination of WC with early life risk factors were very limited. Therefore we stratified BW and WC to investigate their effects on CVD risk.

The present analysis indicated that risk of CVD was highest among those who were smaller at birth and grew up to become adults with high waist circumference. A moderate but consistent association between LBW and cardiovascular risk was found when studying the influence of adult body size and birth size on predicting CVD. Previous studies have demonstrated how growth during infancy, childhood, and adulthood modifies the increased risk of coronary heart disease associated with small body size at birth^3, 20–23^. In a Finland hospital-based study, after age 1 year, rapid weight gain is associated with further increase in risk of coronary heart disease, but only among boys who were thin at birth. In these boys the adverse effects of rapid weight gain on later coronary heart disease are already apparent at age 3 years^21^. Higher body mass index in adulthood is an especially strong risk factor for coronary heart disease among women who were small at birth^4^. Risk of coronary heart disease was especially high for women who crossed from a low centile of weight at birth to a high centile of body mass index in adulthood. In the large cohort of women, size at birth and adiposity in adulthood interacted to predict events of coronary heart disease. WC and BMI were highly correlated, and HWC itself contributed to increased CVD risk significantly^21^. So we choose WC, which reflects abdominal obesity and was considered highly correlated to incident CVD, in our statistical model. Previous studies were mainly conducted on populations who were born before the 1940s. In contrast, the overwhelming proportion of our population was born in or after the 1940s. Our study provide a more contemporary birth cohort to investigate the association of birth weight and waist circumference on cardiovascular disease.

Our findings are limited by several facts. Firstly, our sample size was relatively small and our follow-up rate was relatively low. However, we followed the principle of randomization in baseline enrollment to ensure the representativeness of the sample. The rigorous design and nonselective loss-to-follow-up have greatly reduced the selection bias. Because about one quarter individuals with their BW information at baseline were lost to follow up, we compared the basic characteristics in the participants who attended follow-up with those who were lost, and found no significant differences between the two groups. Secondly, our survey were performed at two time points (baseline and follow up), lacking the data of waist circumference, blood glucose and hypertension evaluation at the period that participants experienced CVD events. In addition, we can’t comment on whether premature birth was the cause of the low birthweight because of lack of the information of gestational age at the time of birth. Moreover, without the information of breastfeeding and birth length, we were unable to analyze the interplay of early growth and CVD risks. However, we demonstrated LBW in the general population could be a predictor of cardiovascular outcome. Finally, as these data are observational, they can only be used to demonstrate a strong association and cannot be interpreted to demonstrate causality.

In conclusion, our findings that birth size and adult adiposity independently predict events of cardiovascular disease have added to the evidence that protection of fetal growth is key in strategies for the primary prevention of CVD. Further benefit will come from preventing abdominal obesity in adulthood, especially for those with low birthweight.

## Method

### Ethics Statement

This study was approved by Ruijin Hospital Ethics Committee (Approval No. 2014–114). Written informed consent signed by each of participants and all methods were carried out in accordance with the relevant guidelines and regulations.

### Study design and Subjects

This cohort was established in 2002 and the design of the study and formation of the study population has been described previously^24, 25^. A cross-sectional survey for the prevalence of type 2 diabetes was conducted in an urban community in Shanghai, China in 2002. A stratified multistage cluster sampling design was employed. First, four (Huoxin, Mingyuancun, Jinsheng, Jiangpu) out of 33 sectors were randomly sampled from the Pingliang community. Then, a sample of 2200 people was randomly selected from 18 000 eligible permanent inhabitants in the four sectors. Valid information was obtained from 2132 people of the sample. All subjects were permanent residents who must live in the community for above 5 years without leaving Shanghai for more than 6 months. There were no serious air pollution, chemical and metal contamination reported in Pingliang community in the past several decades.

The first examination of participants of 2132 men and women aged 18 to 76 years from Pingliang community was conducted from November 2002 to January 2003. At the baseline, all subjects were interviewed with physical examination and standardized questionnaires including information about physician-diagnosed diabetes and hypertension, family history of diabetes, educational background, lifestyle factors, such as cigarette smoking, alcohol consumption and presently used medications for hypertension and diabetes. Plasma glucose was measured during an oral glucose tolerance test (OGTT) and serum lipid profile assayed in all subjects.

The follow-up survey, consisting of 1609 participants, lasted from July to December 2013, but an additional follow-up was made in September to October 2014 for those unavailable at that time^25^. All participants at follow up were interviewed with the same standardized questionnaires with the addition of the information of CVD events.

At baseline, a total of 1010 individuals (389 males and 621 females) provided their birth weight information from birth certification and hospital case records^24^. Among them, 745 participants (283 males and 462 females) were followed (73·8%) and were enrolled in this study for analysis. The mean follow-up time was 10·9 years.

### Birth weight and waist circumference assessment

LBW was defined as BW < 2500 g. WC was measured at the narrowest point below the ribs or halfway between the lowest ribs and the iliac crest in centimeters. Body weight, height and WC were respectively measured to the nearest 0·1 kg and 0·1 cm. HWC was defined as WC ≥ 85 cm in females and WC ≥ 90 cm in males^26^.

### Documentation of end points

We considered CVD (including nonfatal coronary heart disease, nonfatal cerebrovascular disease and cardiovascular death) that occurred between baseline 2002 and October 2014 as major outcomes. Coronary heart disease was further defined as angina pectoris, myocardial infarctions, abnormal coronary arteriography, a cardiac procedure; and cerebrovascular disease includes cerebral ischemic attack, cerebral hemorrhage and cerebral infarction from any cause. Information on deaths was obtained from the official death certificates of the district. In our survey, participants firstly reported their physician-diagnosed CVD and/or medication use during follow up. Then, a physician reviewed their medical records if available. After that, a community staff check the information in the Health Records in this district and a CDC professional confirmed all 83 events in Shanghai Health Records.

### Other variables of interest

Plasma glucose was measured during a 75 g oral glucose tolerance test (OGTT) and type 2 diabetes was defined by a fasting plasma glucose level ≥7.0 mmol/l and/or a 2-h postchallenge glucose level ≥11.1 mmol/l, a previous physician-diagnosed type 2 diabetes, or using antidiabetic medication at baseline. Blood pressure was measured through a mercury sphygmomanometer in sitting position after 5 minutes of rest. Hypertension was defined as the average of the blood pressure values (with a systolic blood pressure (SBP) ≥140 mmHg or diastolic blood pressure (DBP) ≥90 mmHg), or a physician-diagnosed hypertension at baseline. Smoking and alcohol consumption, educational background, diet and physical activity were acquired through well-designed questionnaires. Assessment methods of the above variables have been specifically described elsewhere^24, 25^. Briefly, smoking and drinking status were classified into currently, formerly, and never consumed. Physical activity was calculated as the product of the duration and frequency of each activity (in hours per day) weighted by an estimate of the metabolic equivalent of that activity^25^.

### Statistical analyses

All data were analyzed using SPSS for Windows, Version 18·0 (SPSS, Chicago, IL, USA).

Baseline characteristics were analyzed for significance of differences between groups using one-way analysis of variance (ANOVA) for continuous variables and the chi-squared test for categorical variables. Student’s t-test was applied for comparisons of basic characteristics in those who attended follow-up with those who were lost at follow-up. Binary logistic regression models were used to estimate the adjusted odds ratios (ORs) and 95% confidence intervals (95%CI) for CVD. The analyses were performed adjusting for adult age, sex, diabetes status, hypertension status, lipid profile, WC, smoking and drinking status, physical activity and economic status at baseline in adjusted model. The analyses were carried out to examine the influence of BW and adult WC on cardiovascular disease. Data were expressed as mean ± SD, n (%), or OR (95% CI), all statistical tests were two sided, and a p value of less than 0·05 was considered to be statistically significant.
